# The Potential Contribution of the Health System to Reducing Stunting in SUN Countries

**DOI:** 10.1177/0379572121998127

**Published:** 2021-05-17

**Authors:** Talata Sawadogo-Lewis, Shannon E. King, Tricia Aung, Timothy Roberton

**Affiliations:** 1 1466 Johns Hopkins Bloomberg School of Public Health, Baltimore, MD, USA

**Keywords:** WHA targets, maternal and child nutrition, modeling, stunting, Scaling Up Nutrition, global policy

## Abstract

**Background:**

The global nutrition community has called for a multisectoral approach to improve nutritional outcomes. While most essential nutrition interventions are delivered through the health system, nutrition-sensitive interventions from other sectors are critical.

**Objective:**

We modeled the potential impact that Scaling Up Nutrition (SUN) interventions delivered by the health system would have on reaching World Health Assembly (WHA) stunting targets. We also included results for targets 2, 3, and 5.

**Methods:**

Using all available countries enrolled in the SUN movement, we identified nutrition interventions that are delivered by the health system available in the Lives Saved Tool. We then scaled these interventions linearly from 2012 up to nearly universal coverage (90%) in 2025 and estimated the potential impact that this increase would have with regard to the WHA targets.

**Results:**

Our results show that only 16 countries out of 56 would reach the 40% reduction in the number of stunted children by 2025, with a combined total reduction of 32% across all countries. Similarly, only 2 countries would achieve the 50% reduction in anemia for women of reproductive age, 41 countries would reach at least 50% exclusive breastfeeding in children under 6 months of age, and 0 countries would reach the 30% reduction in low birth weight.

**Conclusions:**

While the health system has an important role to play in the delivery of health interventions, focusing investments and efforts on the health system alone will not allow countries to reach the WHA targets by 2025. Concerted efforts across multiple sectors are necessary.

## Background and Context

The global health community is increasingly focused on malnutrition—undernutrition, micronutrient-related malnutrition, and overweight/obesity. Sustainable Development Goal 2—a continuation of Millennium Development Goal 1—sets the target of ending all forms of malnutrition by 2030.^1,2^ The 65th World Health Assembly (WHA) adopted nutrition-specific targets, setting the global agenda for achieving improved nutritional status throughout the world by the year 2025.^3^ Noting slow progress toward these targets, the 69th WHA then declared 2016 to 2025 to be the United Nations Decade of Action on Nutrition, urging governments to increase their efforts to improve nutrition in their countries.^4^ The 2020 Global Nutrition Report renews this call to action, arguing for the increased integration of nutrition in universal health care to achieve equity in terms of nutrition outcomes.^5^ Leveraging the health system for the delivery of nutrition intervention is also recommended by World Health Organization (WHO)’s Essential Nutrition Actions, and most essential nutrition interventions are indeed delivered through and by the health system.^6,7^

These global efforts—and more specifically the WHA targets—including addressing multiple facets of malnutrition, including overweight, maternal anemia, and birth outcomes. However, the first WHA target is to achieve a 40% reduction of the global number of children under 5 who are stunted. Stunting—being less than 2 *z*-scores below the median on WHO Growth Standards—has both immediate and long-term effects, including reduced adult stature, poor educational performance and achievement, lower lifetime earnings, reduced bone density, and increased risk of cardiovascular disease following weight gain in later life.^8^ Additionally, stunting in childhood increases the likelihood of giving birth to smaller children, which in turn might lead to a separate set of problems for that child.^9,10^ An association has been found between maternal height and grandchild birthweight,^9^ which further highlights the long-term, intergenerational, negative effects of stunting. Because of the long-lasting harmful impact of childhood stunting on individuals as well as society overall, efforts to reduce the number of stunted children are ramping up throughout the world. Stunting is the most common undernutrition metric used in nutrition policies globally.^11^

The Scaling Up Nutrition (SUN) Framework was first published in 2010 as a policy brief by the World Bank, in response to the 2008 Lancet series on undernutrition. Recognizing the need for multisectoral action, the framework outlines the need to integrate nutrition in national strategies for gender equality, agriculture, food security, social protection, education, water supply, sanitation, and health care. Building on these principles, the SUN Road Map outlines how different groups can work together to achieve results. The SUN Movement is now—as of early 2019—a group of 60 countries who have adhered to these principles and are working toward implementing them.^12,13^ Beyond the SUN network, it is commonly recognized that tackling malnutrition requires multisectoral approaches. Nutrition is recognized as a multisectoral problem, requiring a multisectoral solution. In 2018, 80% of WHO member states reported having multisectoral groups or organizations that oversee, coordinate, or harmonize nutrition-related work, such as national nutrition councils, task forces, and advisory bodies.^11^

Despite the recognition that nutrition initiatives must be multisectoral, a 2016 to 2017 WHO global nutrition policy review for 167 member states found that, in most countries, the ministry of health is the governmental entity most commonly tasked with coordinating the implementation of these multisectoral plans and policies. The agriculture and education sectors—followed somewhat distantly by the social welfare sector—are also frequently involved, although not to the same degree. The environment, planning/budget/finance, and trade/industry/labour sectors trail further behind. The health system remains the main delivery channel for nutrition interventions—a finding that holds true for all WHO regions.^11^ While the health sector undoubtedly has a role to play in improving nutrition outcomes, it is unclear how far we will get toward reducing stunting through health-sector interventions alone. If the health system can do it all, such a strategy makes sense. If it cannot, a health system–only approach will be limited, and we should advocate more forcefully for multisectoral efforts. In this paper, we shed light on this question by estimating the potential contribution of nutrition interventions delivered through the health system toward reducing the overall number of stunted children.

## Methods

### Country Selection

We looked at 56 of the 61 countries who have joined the SUN Movement. We carried out the analysis in early 2019, prior to Honduras having joined the movement. We excluded Botswana, Eswatini, Mauritania, and Vietnam because no family planning data more recent than 2010 was available.

### Data

We used the most recent Demographic and Health Surveys (DHS) and Multiple Indicator Cluster Surveys (MICS) for each country as the source of coverage data for health interventions. We took data on Water, Sanitation and Hygiene (WASH) from the WHO-United Nations Children’s Fund Joint Monitoring Program. For unmet need, we used data from the country’s most recent DHS or MICS, except for Sri Lanka, Somalia, Papua New Guinea, and Guinea Bissau where those data were not available. In those cases, we used Family Planning 2020 (FP2020) estimates. Details can be found in supplementary material S1.

### Analysis

We used the Lives Saved Tool (LiST v.5.88) to estimate the changes in stunting that would be achieved by scaling up health-sector interventions. LiST is a mathematical modeling tool which allows users to model the impact of scaling up coverage of maternal, newborn, child health, and nutrition interventions on mortality as well as nutrition outcomes.^14-17^ The model draws from multiple sources to gather estimates for cause specific mortality, intervention coverage, intervention effectiveness, and affected fraction for each intervention separately. Taking stunting as an example, stunting rates are taken directly from household surveys such as DHS and MICS. Some interventions (ie, zinc supplementation) have a direct effect on stunting, whereas other interventions operate through the reduction in diarrhea incidence, which in turn has an effect on stunting (ie, all WASH interventions). Data for the effectiveness of each intervention can be found by clicking on the arrows linking interventions and outcomes on at www.listvisualizer.org.

**Figure 1.**
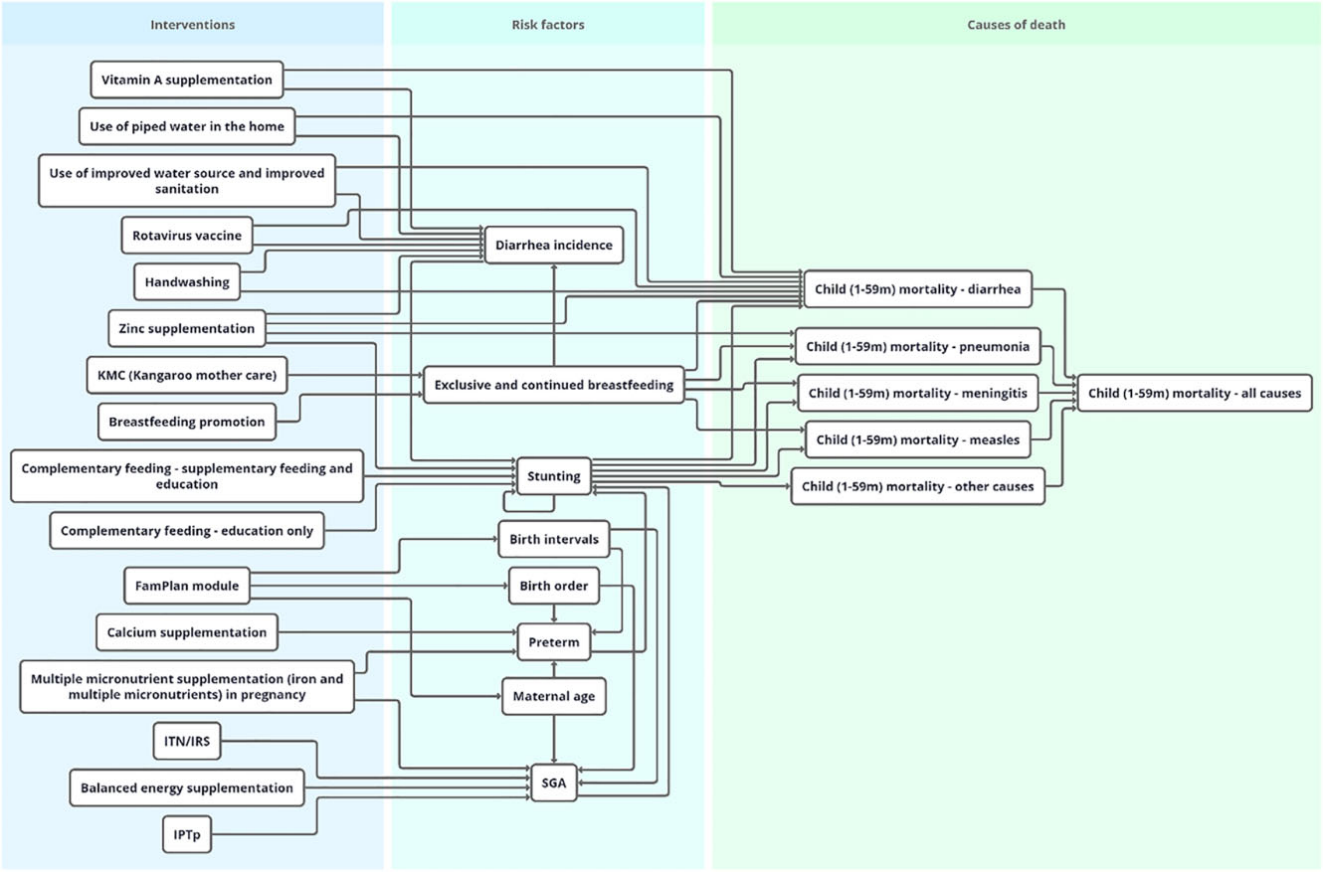
Nutrition interventions in LiST as pictured in the LiST visualizer. LiST indicates Lives Saved Tool.

Lives Saved Tool can model 16 interventions that have an effect on stunting and are delivered through the health system—either directly or operating through risk factors—which are listed in Table 1. Of these, 3 are considered WASH interventions: use of piped water in the home, use of improved water source, and improved sanitation and handwashing. While these WASH behaviors are conducted within the home environment, the health sector often serves are the primary contact point for promoting appropriate WASH behaviors and therefore was included as an intervention within the health system's mandate. Figure 1 shows the links between nutrition interventions, risk factors, and outcomes.

**Table 1.** Nutrition Interventions in Lives Saved Tool (LiST).

Intervention name	Intervention definition
Complementary feeding—supplementary feeding and education	Percentage of mothers intensively counseled on the importance of continued breastfeeding beyond 6 months and appropriate complementary feeding practices and given appropriate dietary supplementation. As a proxy, the percentage of 6- to 23-month-old children receiving all 3 infant and young child feeding (IYCF) practices is used. The 3 IYCF practices refer to continued breastfeeding, appropriate quantity of diet, and appropriate diversity of diet.
Complementary feeding—education only	Percentage of mothers intensively counseled on the importance of continued breastfeeding beyond 6 months and appropriate complementary feeding practices. As a proxy, the percentage of 6- to 23-month-old children receiving all 3 infant and young child feeding (IYCF) practices is used. The 3 IYCF practices refer to continued breastfeeding, appropriate quantity of diet, and appropriate diversity of diet.
Calcium supplementation	Percentage of pregnant women taking 1 g of calcium daily.
Multiple micronutrient supplementation (iron and multiple micronutrients) in pregnancy	Percentage of pregnant women taking a multiple micronutrient supplement daily. A multiple micronutrient supplement is defined as a supplement containing at least iron, folate, and additional vitamins/minerals.
ITN/IRS	Percent of households owning at least 1 insecticide treated bednet (ITN) or protected by indoor residual spraying (IRS).
Balanced energy supplementation	Percentage of pregnant women who are food insecure who receive balanced protein energy supplementation.
IPTp	Percentage of pregnant women receiving 2+ doses of Sp/Fansidar during pregnancy.
Vitamin A supplementation	Percentage of children 6-59 months of age receiving 2 doses of Vitamin A during the last 12 months.
Piped water	Percentage of the population in households with a piped improved drinking water source
Point-of-use filtered water	Percentage of the population in households with point-of-use filtered water with safe storage in the household
Basic sanitation	Percentage of the population in households using an improved sanitation facility (defined as flush or pour flush to piped sewer system, septic tank, or pit latrine; ventilated improved pit [VIP] latrine; pit latrine with slab; or composting toilet), which are not shared
Rotavirus vaccine	Percentage of children 12-23 months who have received 2 or 3 doses of Rotavirus vaccine (according to manufacturer’s schedule).
Handwashing	Percentage of the population living in households with a handwashing facility on premises with soap and water available
Zinc supplementation	Percentage of children 12-59 months of age who are given daily supplements of 10 mg zinc.
KMC (Kangaroo mother care)	Percentage of premature neonates receiving facility-based Kangaroo Mother Care (KMC). KMC is defined as continuous skin-to-skin contact between a mother and her newborn as well as frequent and exclusive breastfeeding.
Breastfeeding promotion	Percentage of children whose mothers receive activities designed to promote breastfeeding. Breastfeeding promotion can either be one-on-one or group meetings.

We created projections from 2019 to 2025 (target year for the WHA objectives). For all interventions except family planning, we scaled up each intervention linearly from current coverage (per DHS/MICS data) to 90% coverage. We chose 90% as a proxy for a “near-universal” coverage level—a threshold that has been used elsewhere for similar analyses.^18,19^ For interventions that were already at higher than 90%, we kept the coverage of that intervention at its current level over the time period. Reductions in the number of stunted children are calculated relative to the 2012 values, as those are the ones which the reductions expressed in the WHA targets refer to.

Given the complexity of establishing a similarly uniform target for meeting family planning needs, we used the total demand met—contraceptive prevalence rate (CPR) and unmet need for family planning combined as per methodology of Alkerma et al^20^—as the “near-universal” coverage level for each country. While CPR is not an intervention per se, the modeling structure in LiST does not allow to model the impact of scaling up individual family planning interventions—which are delivered through the health system and do have an effect on stunting via maternal age, birth order, and birth intervals which in turn affect low birth weight.^21^ We therefore included CPR as a proxy for family planning interventions.

We estimated the number of stunted children for 3 scenarios: if no interventions were scaled up (“No scale up”), if only CPR was scaled up to meet unmet need (“Only CPR”), and if all interventions including CPR were scaled up (“All interventions”). Results by country are available in supplementary material S1.

Using the “All interventions” projections, we created for tables for the other WHA targets available in LiST, namely reducing maternal anemia by 50% in women of reproductive age, reducing low birth weight by 30%, increasing the rate of exclusive breastfeeding in for first 6 months to at least 50%, and reducing or maintaining wasting to less than 5%.^3^ Results by country are available in supplementary material S1. We present results comparing to the 2012 values, as the targets refer to reductions compared to the 2012 data.

## Results

### Number of Stunted Children

**Figure 2.**
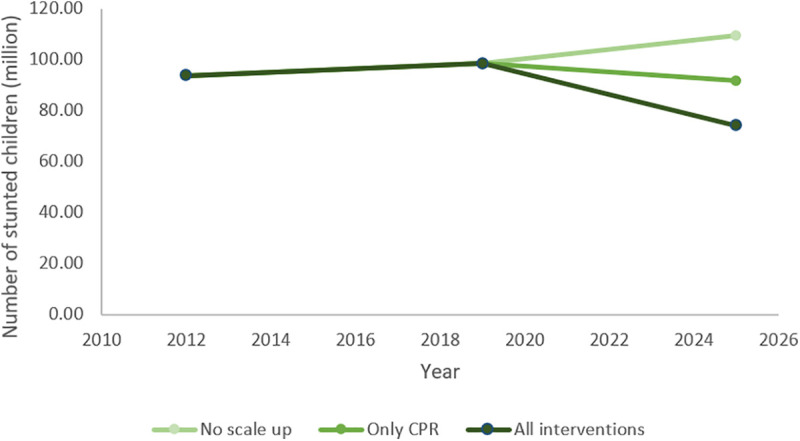
Number of stunted children and potential relative decrease for 3 scenarios: no scale up, only contraceptive prevalence scaled up, and all intervention scale up scenarios.

Figure 2 shows the number of children stunted in 2025 in 3 different scenarios. In the “No scale up” scenario, the highest number of children are stunted in 2025. In the “Just CPR” example, the reduction in the number of stunted children is driven mostly by the reduction in fertility (ie, reducing the number of children overall), along with higher maternal age and increased birth spacing. For “All interventions,” which shows the highest impact, all interventions including meeting unmet need for contraception are scaled up. We present data from 2012 to 2019 for reference only because WHA targets refer to the 2012 values. For all other tables in this paper, we only present results pertaining to the “All interventions” scenario.

### Impact of Scale Up on Stunting

Only 16 of the 60 countries (highlighted in color in Table 2) would reach the WHA target of a 40% percent decrease in the number of stunted children, if they were to scale up health-sector interventions to near-universal levels. The majority of countries do not reach the target. Taken as a whole, we estimate a 32% total reduction of stunted children across the 56 countries.

**Table 2.** Impact of Scale Up of All Interventions on the Number of Stunted Children, by Country.

Number of stunted children				% decrease
Country	% children stunted in 2012	2012	2025	
Afghanistan	37.3	1 972 266	1 838 968	−30.18%
Bangladesh	41.88	6 381 743	3 846 043	−37.28%
Benin	35.92	590 327	359 ,666	−55.99%
Botswana	36.01	94 625	75 225	−18.88%
Burkina Faso	34.39	1 034 750	861 834	−32.66%
Burundi	56.38	994 269	781 671	−36.39%
Cambodia	35.55	638 672	481 282	−23.69%
Cameroon	31.93	1 166 743	962 922	−25.96%
Central African Republic	40.92	300 068	279 883	−21.43%
Chad	38.75	964 572	958 684	−33.63%
Comoros	30.87	34 658	24 003	−42.34%
Congo	23.31	174 341	115 303	−48.50%
Costa Rica	22.07	78 097	40 160	−46.83%
Côte d’Ivoire	28.19	969 440	746 691	−39.50%
Democratic Republic of the Congo	42.41	5 538 841	5 204 004	−34.12%
El Salvador	15.28	93 476	40 195	−44.41%
Ethiopia	42.54	6 267 256	5 519 976	−32.76%
Gabon	16.96	43 660	29 917	−42.60%
Gambia	24.25	82 655	61 090	−39.04%
Ghana	21.36	806 266	549 001	−44.23%
Guatemala	47.05	935 621	626 622	−30.99%
Guinea	30.56	559 889	479 205	−31.54%
Guinea-Bissau	32.3	87 224	59 571	−47.12%
Haiti	22.06	286 252	137 475	−57.89%
Indonesia	37.52	9 215 440	5 534 276	−32.29%
Kenya	28.8	2 017 279	1 169 314	−42.45%
Kyrgyzstan	17.59	125 822	108 226	−26.92%
Lao People’s Democratic Republic	42.15	330 662	241 238	−28.54%
Lesotho	34.53	84 913	36 244	−42.03%
Liberia	32.76	219 850	153 138	−35.56%
Madagascar	49.9	1 757 930	1 495 312	−25.39%
Malawi	43.03	1 184 729	747 770	−44.07%
Mali	36.3	1 126 469	988 652	−35.11%
Mauritania	29.6	174 075	140 622	−35.80%
Mozambique	42.8	1 877 968	1 470 212	−31.98%
Myanmar	28.82	1 394 151	826 305	−29.21%
Namibia	23.26	70 143	43 165	−42.81%
Nepal	39.03	1 117 043	1 068 199	−15.19%
Niger	43	1 591 522	1 829 915	−26.71%
Nigeria	35.79	10 519 105	9 428 251	−27.40%
Pakistan	44.14	11 097 435	9 441 209	−30.34%
Papua New Guinea	37.36	388 466	337 205	−28.55%
Peru	18.33	537 384	301 390	−41.54%
Philippines	32.62	3 758 136	2 799 185	−31.66%
Rwanda	40.48	728 494	467 986	−36.98%
Senegal	18.76	423 117	307 609	−38.07%
Sierra Leone	39.41	431 824	294 908	−30.57%
Somalia	41.02	987 548	1 035 322	−27.15%
South Sudan	29.42	492 817	471 533	−23.88%
Sri Lanka	37.67	670 086	430 710	−14.53%
Sudan	36.29	2 058 782	1 906 518	−24.85%
Swaziland	28.09	45 710	30 593	−27.31%
Tajikistan	25.84	303 664	209 137	−37.68%
Togo	27.99	307 833	202 069	−43.28%
Uganda	32.09	2 142 535	1 614 069	−40.24%
Tanzania	38.79	3 225 610	2 695 966	−34.24%
Viet Nam	22.12	1 647 254	812 221	−38.78%
Yemen	46.1	1 757 557	1 337 267	−34.06%
Zambia	40.14	1 058 126	784 511	−37.37%
Zimbabwe	29.68	638 395	253 000	−38.84%
**Total**		**95 603 587**	**75 092 641**	−**32.36%**

### Impact of Scale Up on Achieving WHA Targets on Anemia, Low Birth Weight, Breastfeeding, and Wasting

According to our estimates, only 2 countries are likely to reach a 50% reduction in anemia by 2025. For target 2, no country is on track to reach the 30% reduction in low birth weight. For target 5, the projections are more encouraging with 41 countries out of 60 reaching at least 50% of children under the age of 6 months exclusively breastfed.

Similarly, 42 countries reach target 6 of having a prevalence under 5% for wasting in 2025. Importantly for this target, the interventions scaled up do not include interventions that focus specifically on wasting (specifically, either severe acute malnutrition management (SAM) or moderate acute malnutrition management (MAM)).

## Discussion

While there are increasing efforts and momentum to integrate nutrition better into the health system,^22^ our findings show that while the health system can make important contributions to the reduction in number of stunted children, if not supported by other sectors, even at near full-capacity, the health system’s contribution alone is off-track from being able to reach the 2025 WHO stunting target.^23^ This is particularly pronounced in African countries which also have the highest number of stunted children in our sampled countries. Health sector interventions alone would also not achieve WHA targets 2, 3, and 5—our analysis does not appropriately capture the potential effect for 6.

The SUN movement recognizes the importance of establishing meaningful multi-stakeholder platforms (MSPs). Steps 1 to 3 of SUN’s 8-step theory of change focus on establishing and leveraging these MSPs,^24^ indicating that collaborating with multiple stakeholders is recognized as key for the SUN movement. A 2018 midterm review of SUN countries however found that while some multisectoral engagement has happened in SUN countries, it has been “more akin to a faucet rather than a stream.”^25^ Our findings support increasing efforts for a genuinely multisectoral approach, given that even if operating at its maximum capacity, the health system as a silo would not be able to reach goals.

With only 5 years left to reach global nutrition targets, there has been increased attention to funding for nutrition.^5,26,27^ Key themes of the upcoming Nutrition for Growth Summit—the largest financial and political pledging event for nutrition—are investing in nutrition as part of health systems and food systems.^28^ It is a critical time to advocate for multisectoral approaches toward funding nutrition since investing in health systems alone will not be sufficient to achieve WHA targets.

Khalid et al^26^ suggest that increasing investments in nutrition-sensitive interventions would have an association with decreases in childhood stunting rates, but that the effect would be lagged by 3 to 4 years. They also found that this association would be stronger in countries with higher burdens of malnutrition.^26^ Our findings align with this conclusion, insofar as we also observe the highest impact in countries with the higher burden of malnutrition. Monteiro et al attributed two-thirds of the reduction in childhood stunting in Brazil from 1996 to 2007 to 4 elements, namely: maternal schooling (21.7%), family purchasing power (25%.7), maternal and child health care (11%), and coverage of water supply and sanitation services (4.3%).^29^ Indeed, these findings support that while the health system can contribute to the reduction of stunting, nutrition-sensitive interventions play a crucial and large role as well.

**Table 3.** Summary of WHA Targets and the Potential Contribution of Health System Interventions to Achieving Targets.

WHA target	Indicator	Target	Average achievement across countries if all health-sector interventions scaled-up	Number of countries achieving the target
Target 1	Prevalence of low height-for-age in children under 5 years of age	Achieve a 40% reduction in the number of children under 5 who are stunted	32.36% reduction	16/60
Target 2	Prevalence of hemoglobin <11 g/dL in pregnant women Prevalence of hemoglobin <12 g/dL in nonpregnant women	Achieve a 50% reduction of anemia in women of reproductive age	23.13% reduction	2/60
Target 3	Prevalence of infants born <2500 g	Achieve a 30% reduction in low birth weight	9.22% reduction	0/60
Target 5	Prevalence of exclusive breastfeeding in infants aged 6 months or less	Increase the rate of exclusive breastfeeding in the first 6 months up to at least 50%	N/A^a^	41/60
Target 6	Prevalence of low weight-for-height in children under 5 years of age	Reduce and maintain childhood wasting to less than 5%	N/A^a^	42/60

Abbreviation: WHA, World Health Assembly.

Three WASH interventions are responsible for relatively high numbers of stunting cases averted namely: hand washing with soap (4%), basic sanitation (5%), and piped water (12%). In LiST’s model, as of 2020, all 3 affect diarrhea incidence which in turn affects rates of stunting. This approach is supported by a 2017 systematic review,^30^ using the then-most recent evidence available. However, the effectiveness of WaSH interventions has recently come under scrutiny, with some studies suggesting a smaller effectiveness^31,32^ and other studies suggesting greater effectiveness.^33^ We therefore caution readers in their interpretation of these results.

## Limitations

We recognize that using total demand for family planning met as CPR and unmet need for family planning may have underestimated the cases of stunting avoided through the decrease in children born with a low birth weight. Additionally, given insufficient data for inclusion, the link between malaria and stunting is not currently captured by LiST^34^ and therefore not included in these calculation. We additionally recognize that LiST itself relies on many estimates, and therefore, some uncertainty has been introduced into our results. Additionally, stunting data from household surveys have large uncertainty,^35^ and it is difficult to obtain accurate measurements of other nutrition indicators such as low birth weight.^36^ Nevertheless, these are data that are available in household surveys, and the best estimates available at this time. We have also modeled the impact of scaling interventions up from 2019 onward (using the most recent available data as baseline, to reflect the reality), rather than from the initial target date of 2012. The modeled impact would have been greater if we had begun the projection in 2012. The difference between the models (one starting in 2019 and other in 2012) represents the missed opportunity corresponding to 7 years of scale up.

We also performed this analysis prior to the COVID-19 pandemic. Early estimates of the effects of the pandemic—both on health systems generally and on nutrition specifically—suggest that the impact will be substantial.^37^ Food systems in many settings were already fragile prior to the pandemic, and the repercussions of the pandemic have increased the magnitude of undernourished populations.^5^ We therefore believe that the numbers presented in this paper underestimate how far off track countries are likely to be for reaching the 2025 targets.

## Conclusion

Our findings suggest that health systems can have an impact in reducing stunting, but even if they were to contribute at maximum capacity, most countries would not reach stunting targets—or most other nutrition targets. To reach the WHO stunting target and to reduce the impact of stunting on children’s health and societies’ well-being more generally, countries need to continue efforts toward ensuring all sectors contribute significantly.

## Supplemental Material

Supplemental Material, sj-xlsx-1-fnb-10.1177_0379572121998127 - The Potential Contribution of the Health System to Reducing Stunting in SUN Countries Click here for additional data file. Supplemental Material, sj-xlsx-1-fnb-10.1177_0379572121998127 for The Potential Contribution of the Health System to Reducing Stunting in SUN Countries by Talata Sawadogo-Lewis, Shannon E. King, Tricia Aung and Timothy Roberton in Food and Nutrition Bulletin
